# Identification of Dof transcription factors in *Dendrobium huoshanense* and expression pattern under abiotic stresses

**DOI:** 10.3389/fgene.2024.1394790

**Published:** 2024-04-22

**Authors:** Fangli Gu, Wenwu Zhang, Tingting Wang, Xiaomei He, Naifu Chen, Yingyu Zhang, Cheng Song

**Affiliations:** ^1^ Anhui Engineering Laboratory for Conservation and Sustainable Utilization of Traditional Chinese Medicine Resources, College of Biological and Pharmaceutical Engineering, West Anhui University, Lu’an, China; ^2^ College of Life and Health Sciences, Anhui Science and Technology University, Fengyang, China; ^3^ The First Affiliated Hospital, College of Clinical Medicine of Henan University of Science and Technology, Luoyang, China

**Keywords:** *Dendrobium huoshanense*, DNA binding with one finger, phylogeny, abiotic stress, bioinformactics analysis

## Abstract

**Introduction:** DNA-binding with one finger (Dof) transcription factors (TFs) are a unique family of TFs found in higher plants that regulate plant responses to light, hormones, and abiotic stresses. The specific involvement of *Dof* genes in the response to environmental stresses remains unknown in *D. huoshanense*.

**Methods:** A total of 22 *Dof* family genes were identified from the *D. huoshanense* genome.

**Results:** Chromosome location analysis showed that *DhDof* genes were distributed on 12 chromosomes, with the largest number of *Dof* genes located on chromosome 8. The phylogenetic tree revealed that DhDofs could be categorized into 11 distinct subgroups. In addition to the common groups, DhDof4, DhDof5, DhDof17, and the AtDof1.4 ortholog were clustered into the B3 subgroup. Group E was a newly identified branch, among which DhDof6, DhDof7, DhDof8, and DhDof9 were in an independent branch. The conserved motifs and gene structure revealed the differences in motif number and composition of DhDofs. The dof domain near the N-terminus was highly conserved and contained a C_2_-C_2_-type zinc finger structure linked with four cysteines. Microsynteny and interspecies collinearity revealed gene duplication events and phylogenetic tree among *DhDofs*. Large-scale gene duplication had not occurred among the *DhDofs* genes and only in one pair of genes on chromosome 13. Synteny blocks were found more often between *D. huoshanense* and its relatives and less often between *Oryza sativa* and *Arabidopsis thaliana*. Selection pressure analysis indicated that *DhDof* genes were subject to purifying selection. Expression profiles and correlation analyses revealed that the *Dof* gene under hormone treatments showed several different expression patterns. *DhDof20* and *DhDof21* had the highest expression levels and were co-expressed under MeJA induction. The *cis*-acting element analysis revealed that each *DhDof* had several regulatory elements involved in plant growth as well as abiotic stresses. *q*RT-PCR analysis demonstrated that *DhDof2* was the main ABA-responsive gene and *DhDof7* was the main cold stress-related gene. IAA suppressed the expression of some *Dof* candidates, and SA inhibited most of the candidate genes.

**Discussion:** Our results may provide new insights for the further investigation of the *Dof* genes and the screening of the core stress-resistance genes.

## Introduction

Dof is a type of plant-specific TFs that regulate gene expression by binding to promoters or interacting with specific proteins, and play a crucial role in regulating a wide range of plant physiological functions (Gupta et al., 2018). The N-terminus of Dof protein shares a highly conserved Dof domain consisting of 52 amino acids. The core motif is covalently combined with Zn^2+^ to form a single zinc finger structure, which specifically binds to promoter sequences with the core [T/AAAAG] motif in the downstream gene (Gupta et al., 2015). The C-terminus harbors a transcriptional regulatory domain with diverse functionalities, enabling its interaction with various regulatory proteins and selective activation of gene expression. The amino acids in the domain are poorly conserved and vary greatly between different Dof members, which in turn leads to differences in Dof protein functions (Ma et al., 2015). Many *Dof* members have been found in higher plants and *ZmDof1* was first found in *Zea mays* (Lijavetzky et al., 2003; Cai et al., 2013; Ma et al., 2015; Song et al., 2016; Song et al., 2022). In monocot plants, *Eleusine coracana* (48 individuals), *Musa acuminata* (74 individuals), and *Setaria italica* (35 individuals) had been identified (Dong et al., 2016; Zhang et al., 2017; Gupta et al., 2018). In dicotyledonous plants, *A. thaliana* (39 individuals), i (60 individuals), *Populus trichocarpa* (41 individuals), and *Betula platyphylla* (26 individuals) had been identified (Le Hir and Bellini, 2013; Wang et al., 2017; Zhang et al., 2017; Sun et al., 2021).

Dof TFs are involved in light response (Park et al., 2003; Ward et al., 2005), photoperiod regulation (Fornara et al., 2009; Corrales et al., 2014), sugar metabolism (Tanaka et al., 2009), nitrogen metabolism (Yanagisawa et al., 2004), seed development (Papi et al., 2002), cell cycle regulation (Xu et al., 2016), abiotic stresses (Zang et al., 2017), and other complex physiological processes (Zhuo et al., 2020). *DAG1* (*DOFAFFECTING GERMINATION 1*) mutant was sensitive to red light and regulated by phytochrome B. It could reduce the red light and GA synthesis during seed germination in *A. thaliana* (Gabriele et al., 2010). *OBP1* (*OCS element binding factor binding protein 1*) could shorten cell division cycle and cause dwarf plants (Skirycz et al., 2008). *AtOBP3* was affected by SA and auxin. As a downstream regulator of phytochrome B, *AtOBP3* is regulated by cryptochrome 1 (Ward et al., 2005). *AtDof5.4/OBP4* acts as a negative regulator to regulate cell expansion and cell cycle progression. *AtOBP4* inhibited cell growth and proliferation and caused obvious defects such as dwarfing growth and fewer flowers (Luo et al., 2022). *CDFs* (*CYCLING DOF FACTOR*) are widely involved in photoperiod regulation, and overexpression of the *AtCDF1* gene showed an early flowering phenotype (Goralogia et al., 2017). *SlCDF* was not only involved in the regulation of photoperiod in tomato but also enhanced plant tolerance under abiotic stresses such as drought and salinity (Xu et al., 2021). *AtDof2.4* and *AtDof5.8* were involved in the formation of rosette leaf veins and flower bud vascularity (Konishi and Yanagisawa, 2007; Noguero et al., 2013). *AtDof6* negatively regulated seed germination in the ABA hypersensitivity plants and increased the expressions of *ABA1* and associated genes. *AtDof5.6/HCA2* participated in the formation of the interfascicular cambium and vascular tissue development. *AtDOF4.7* regulates abscission by directly controlling the transcription of cell wall hydrolases. *AtDof4.7* gene was highly expressed in the siliques and inner layers of *A. thaliana*. *AtDof2.1* sped up JA-stimulated senescence via the MYC2-AtDof2.1-MYC2 feed-forward loop, and promoted leaf senescence (Zhang et al., 2022).

Numerous studies have demonstrated that Dof TFs have a role in plant resistance responses to abiotic stress (Song et al., 2024). The expression of *AtDof1.1* was stimulated by MeJA, resulting in a 2-3-fold increment in expression level (Skirycz et al., 2006). *AtDof5.8* regulated the plasma membrane-bound *NAC* gene *ANAC069*, which contributed to the response to salt stress (He et al., 2015). High salt, drought, high temperature, and ABA all increased the expression of the *AtCDF3* gene. Overexpression of *AtCDF3* improved drought, low temperature, osmotic stress and shortened flowering time in transgenic *A. thaliana*. *SlCDF1*, the *CDF* homolog of *A. thaliana*, increased in expression to different levels when exposed to drought, salt, heat, and low temperatures. Overexpression of *SICDF1* and *SICDF3* in *Arabidopsis* improved the drought resistance of transgenic plants (Liu et al., 2023). The transgenic cotton overexpressing *GhDof1* exhibited much greater salt and cold tolerances compared to the wild-type plants. Salt stress promoted the growth of the root system in *GhDof1*-overexpressing plants. The expressions of *GhP5CS*, *GhSOD*, and *GhMYB* in the transgenic lines was upregulated to varying degrees (Su et al., 2017). In *Tamarix bristle*, *ThDof1.4* significantly improved the abiotic stress tolerance of transgenic plants by increasing proline content, ROS scavenging, and the expression of *ThSOD* and *ThP5CS* genes (Li et al., 2022). *TaDofs* were involved in wheat grain development and abiotic stress responses. *TaDof16*, *TaDof26* and *TaDof96* were significantly upregulated under drought stress (Liu et al., 2020). Thirty-three *Dof* genes were identified in pepper. The temporal and pathogen-specific differences under biotic stress were discovered in *CaDofs*, which demonstrated the functional diversity of *CaDofs* (Kang et al., 2016).

Dof transcription factors play a certain role in the regulation of primary metabolism and secondary metabolism. During carbon metabolism, Dof transcription factor regulates the expression of its related genes. In maize, ZmDof1 can bind to the AAAG motif in the promoter region of *OsCS4PPDK* to increase the expression of the *C4 phosphoenolpyruvate carboxylase* and *pyruvate kinase* genes in the cytoplasm; however, ZmDof2 inhibits the *C4 phosphoenolpyruvate carboxylase* gene expression (Yanagisawa, 2002). In sweet potatoes, overexpression of the *SRF1* gene in the roots significantly reduces the transcription of the *βfruct2* gene, thereby reducing the accumulation of sucrose invertase. This leads to a reduction in the concentration of monosaccharides and increases the starch content in the tubers, thereby regulating carbon metabolism (Tanaka et al., 2009). Dof protein can not only regulate carbon metabolism but also improve plant nitrogen utilization and increase nitrogen content. Once overexpressing the *ZmDof1* gene in Arabidopsis, the nitrogen content of positive plants increased, and they were able to grow well under low-nitrogen conditions. Overexpression of *OsDof25* in *Arabidopsis thaliana* promotes the expression of high-affinity and low-affinity ammonium transporter *AtAMT1.1* and *AtAMT2.1* and inhibits the expression of high-affinity nitrate transporter *AtNRT2.1*. The expression of *kinase, phosphoenolpyruvate carboxylase*, *NADP-dependent* and *NAD-dependent isocitrate dehydrogenase* genes is increased. Dof transcription factor is related to two secondary metabolic processes: the phenylpropionic acid synthesis pathway and the flavonoid synthesis pathway. Overexpression of *AtDof4.2* can increase the sensitivity of plants to light at low temperatures. At low temperatures and strong light, AtDof4.2 can negatively regulate the synthesis of flavonoids and positively regulate the synthesis of cinnamic acid (Song et al., 2022). Dof transcription factors can also regulate lipid synthesis and control fatty acid content. Acetyl-CoA carboxylase and long-chain fatty acyl-CoA synthase are two key enzymes in the lipid synthesis process. GmDof4 and GmDof11 specifically bind to the promoters of the *acetyl CoA carboxylase* and *long-chain CoA synthase* genes, respectively (Dong et al., 2016).

Wild varieties of *D. huoshanense* have been plundered in large quantities over the past decade, and the original species is now threatened (Song et al., 2020). Semi-shade, moisture, and a particular environment around stones and moss are required for their fast growth (Song et al., 2021). There are lots of TFs that have been found to regulate abiotic stress and secondary metabolism in *Dendrobium spp.* (Song et al., 2022). bHLH TFs play a positive role in JA signaling cascade and abiotic stresses (Jiao et al., 2022). A genome-wide identification and analysis of WRKY TFs was employed and screened out several hormone- and cold stress-responsive genes (Zhang et al., 2023). Here, the identification, comparative genomics, and expression analysis of *Dof* genes were conducted in *D. huoshanense*. A total of 22 *Dof* genes were identified, which can be divided into 11 subgroups based on the homologous genes. Conserved motif and gene structure analysis indicated that these Dof proteins shared a common Dof domain, which contained a representative zinc finger structure through multiple sequence alignments. Dof members on the same evolutionary branch have similar motif compositions, but the structural composition of exons and introns is quite different. Microsynteny analysis revealed that the generation of *DhDofs* were not derived from large-scale gene duplication but mainly came from proximal and dispersed duplication, and these *DhDof* genes have undergone purifying selection. Comparative transcriptome analysis revealed that *DhDof20* and *DhDof21* are the main JA-responsive genes. The *cis*-acting element analysis revealed that the promoter of *DhDof* has many elements related to hormone response, abiotic stress, photoperiod, and vegetative growth. Expression pattern analysis showed that *DhDof2* is the key ABA-responsive gene and *DhDof7* is the main cold stress-related gene.

## Materials and methods

### Sample collection and conditions

*D. Huoshanense* materials were collected at the Plant Cell Engineering Center of West Anhui University (Luan, China). The culture conditions were 25°C ± 2°C, with a 12 h day and 12 h dark cycle (Zhang et al., 2023). MS medium (without hormone addition) was used for the tissue culture of seedlings. When the seedlings grow to a height of 5–8 cm in subculture, hormone treatments with different concentrations are added. A 100 mol/L ABA solution was supplied to the medium, and the expression levels of *Dof* genes were measured on days 0, 2, and 4. The samples were treated with a 50 mol/L MeJA solution and collected at 0, 2, and 8 days. The expression level of the *Dof* genes was measured on days 0, 2, and 4 after adding the 0.1 mg/L IAA solution and the 100 mol/L SA to the medium. After the application of the 0.5 mmol/L GA solution to the plants, samples were collected on days 0, 3, and 6 to measure the expression of *Dof* genes. During the low-temperature treatment, the samples were first stored at a temperature of 4°C in a refrigerator. To measure the expression of *Dof* genes, samples were obtained on days 0, 1, and 4.

### Identification, physical properties and chromosomal location analysis of *Dof* gene

The genome sequence and annotation of *D. huoshanense* were obtained from the National Center for Biotechnology Information database (accession: PRJNA597621). The Hidden Markov Model (HMM) of the Dof domain (PF02701) is downloaded from the InterPro database. The Arabidopsis Dof homologs were downloaded from the Arabidopsis Information Resource (https://www.arabidopsis.org/), and the Blastp method was used for sequence alignment to obtain non-redundant Dof genes. Then, Pfam (http://pfam-legacy.xfam.org/), InterPro (http://www.ebi.ac.uk/interpro/), and SMART (https://smart.embl.de/) were applied for the verification of the Dof candidates. Using genome and gff annotations, chromosomal location analysis of 22 *DhDof* genes was completed, and then TBtools software was used to perform visual analysis of *Dof* genes (Chen et al., 2020). By using the Expasy web service (http://expasy.org/), it was possible to predict the molecular weight, isoelectric points, instability index, aliphatic index, and hydropathicity. The Plant-mPLoc plug-in of Cell-PLoc 2.0 software (http://www.csbio.sjtu.edu.cn/bioinf/Cell-PLoc-2/) was used for the subcellular localization prediction.

### Phylogenetic tree analysis of Dof proteins

To determine the composition and classification of DhDof, a phylogenetic tree of the two species was constructed using the Arabidopsis nomenclature. MEGA (v.6.0.6) software was used to construct the evolutionary tree using the neighbor-joining method. First, ClustalW software was used to perform protein sequence alignment with the Poisson model and pairwise deletion mode. The bootstrap test was set at 1,000 replicates. IQ-TREE (v.1.6.12) software was used to construct the evolutionary tree using the maximum likelihood method. First, IQ-TREE was used to calculate the optimal model of these Dof sequence alignment files. “VT + F + R5” was chosen as the best-fit model under the Bayesian information criterion. Then, using this model and the “iqtree.exe -s./bidui.fas -m VT + F + R5 -bb 1000 -alrt 1000 -nt AUTO” command, we constructed an unrooted consistency evolutionary tree. The two evolutionary trees passed the identity (>40%) and SH-aLRT support (>80%)/ultrafast bootstrap support (>95%) thresholds to be manually classified.

### Conserved domain, motif and exon/intron analysis of *Dof* genes

The conserved domain database (https://www.ncbi.nlm.nih.gov/Structure/bwrpsb/bwrpsb.cgi) was used for domain searches of Dof proteins. The MEME-suite web service (http://meme.nbcr.net/meme/cgi-bin/meme.cgi) was applied for motif searching and the result was visualized using TBtools software (https://github.com/CJ-Chen/TBtools). Motifs identified by MEME were further retrieved in the InterPro database (http://www.ebi.ac.uk/interpro/). The gene structure was performed using Gene Structure Display Server (http://gsds.cbi.pku.edu.cn/) and visualized by TBtools.

### Multiple sequence alignment and amino acid composition analysis of Dof proteins

ClustalW was used to align DhDof protein sequences first, and then the alignment files were imported into GeneDoc software (http://psc.edu/biomed/genedoc) for visualization of protein sequences. Black background, gray background, and white background represent different matching values of amino acids. WebLogo 3 software (https://weblogo.threeplusone.com/create.cgi) was used to visualize the sequence logo of the Dof domain.

### Comparative genomic analysis of *Dof* genes

The MCScanX software (https://github.com/wyp1125/MCScanX) was applied to examine collinearity between *D. huoshanense* and the other four species. The genome sequences and annotations of *D. nobile, D. chrysotoxum, O. sativa* and *A. thaliana* are downloaded from NCBI (accession: PRJNA725550), NCBI (accession: PRJNA664445), Ensembl Plants (genome assembly: IRGSP-1.0), and TAIR (genome assembly: TAIR10). The microsynteny circus of the gene duplication event was visualized by TBtools software. Then, each *Dof* sequence of *D. huoshanense, D. nobile, D. chrysotoxum, A. thaliana,* and *O. sativa* was aligned against itself using BLASTp with an E-value threshold of e^−10^ to obtain the syntenic blocks between each two species.

### C*is*-acting element analysis of *Dof* genes

The 2 kilobase (kb) regions flanking the promoters were obtained from the genomic sequences. The *cis*-acting elements in *Dof* promoters were obtained from the PlantCARE database (http://bioinformatics.psb.ugent.be/webtools/plantcare/html/). TBtools software was utilized to visualize the basic elements associated with growth, phytohormones, and environmental responses.

### Expression profiling of *Dof* genes

The previous transcriptome datasets (https://bigd.big.ac.cn/gsa/browse/CRA006607) were used to compute and normalize the expression of Dof genes as fragments per kilobase per million mapped fragments (FPKM). The sample reads were aligned to the *D. huoshanense* reference genome using the HISAT software, which could be available at https://daehwankimlab.github.io/hisat2/. The FPKM values for mRNA expression analysis were computed using StringTie (https://github.com/gpertea/stringtie). Salmon was employed to quantify expression levels from RNA-seq data (https://combine-lab.github.io/salmon/). The heatmap construction utilizes the normalized FPKM values (log_2_ (FPKM)) of the unigenes.

### *q*RT-PCR analysis of *Dof* genes

RNA was isolated and extracted using the RNA extraction reagent (QIAGEN, Germany) according to the manufacturer’s protocol. The genomic DNA (gDNA) was cleaved using the RNAse-Free DNase Set from QIAGEN, a company based in Germany. The RNA samples were assessed for purity using the NanoDrop 2000c (Thermo Fisher Scientific, United States) and gel electrophoresis. The reverse transcription PCR was performed using the PrimeScriptTM II 1st Strand cDNA Synthesis Kit from TaKaRa, a company based in China. The SYBR Premix Ex TaqTM II, manufactured by TaKaRa in China, was employed for quantitative reverse transcription polymerase chain reaction (*q*RT-PCR) on a 7,500 series real-time fluorescence quantitative cycler manufactured by Bio-RAD in the United States of America. The primers for the *q*RT-PCR test were prepared using the Primer Premier 5.0 software. The *ACTIN* gene served as the reference gene (Song et al., 2021). The primers utilized for *q*RT-PCR analysis are listed in Supplementary Table S2. Each experiment was repeated three times in triplicate, and a total of three biological replicates were undertaken. The gene expression levels were determined using the 2^-△△CT^ method.

## Results

### Chromosome location and phylogenetic analysis of *Dof* genes

**FIGURE 1:**
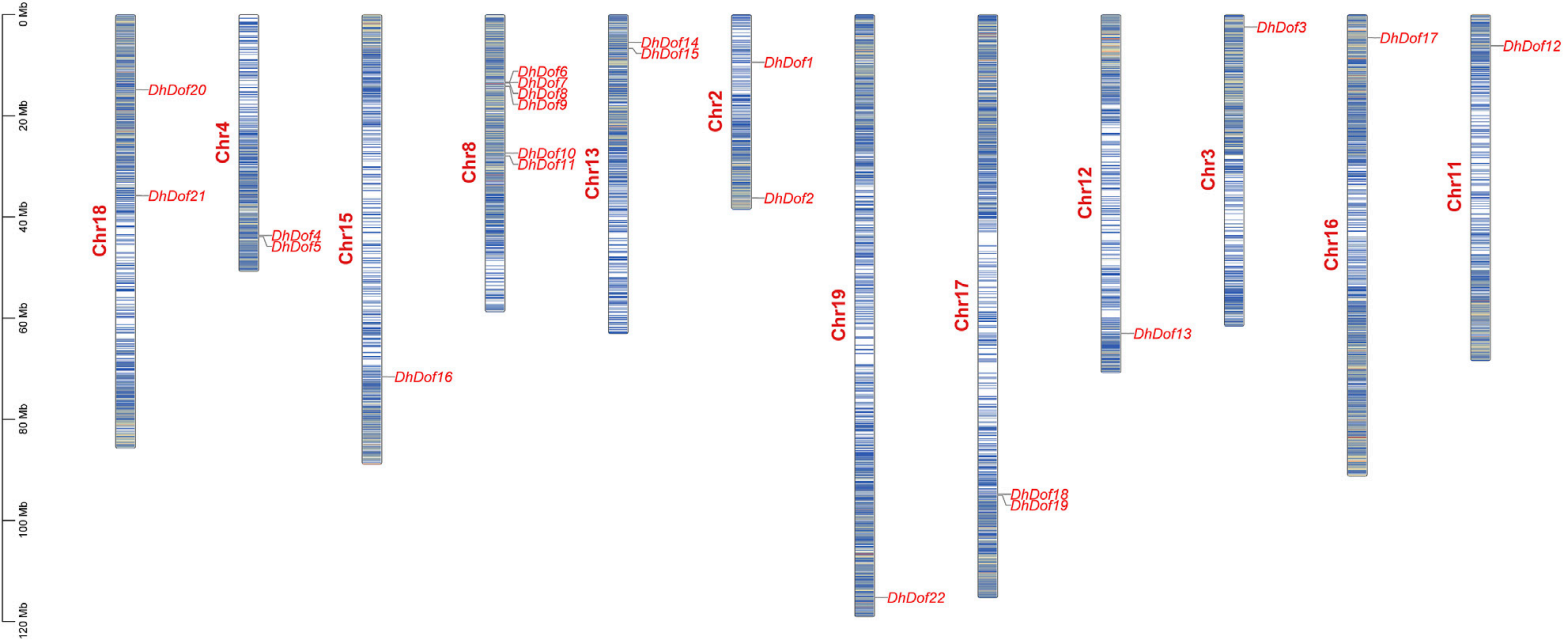
Chromosomal location analysis of the *DhDof* genes.

**FIGURE 2:**
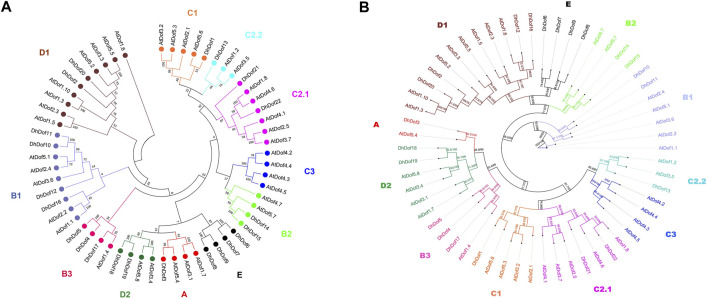
Phylogenetic tree of Dof proteins from *D. huoshanense* and *A. thaliana*. **(A)** Neighbor-joining method; **(B)** Maximum-likelihood method.

A total of 22 *Dof* genes were screened from *D*. *huoshanense* genome, and their conserved domains were further compared and verified through Pfam, InterPro, and SMART databases. Using the genome and annotation files, the chromosomal locations of these *Dof* genes were determined. The original *Dofs* were renamed based on their order and location on different chromosomes. The results showed that 22 *Dof* genes were dispersed on 12 chromosomes (Figure 1). The *Dof* genes anchored on chromosome 8 genetically formed a cluster at a physical distance. However, there was only one *Dof* gene on chromosomes 11, 12, 13, 15, 16, and 19. To clarify the evolutionary relationships and classification of these DhDofs, the phylogenetic tree was initially built using the neighbor-joining method (Figure 2A). According to the branch value and the default classification, DhDof proteins were categorized into 5 groups and 11 subgroups, namely, A, B1, B2, B3, C1, C2.1, C2.2, C3, D1, D2, and E. Among them, DhDof4, DhDof5, DhDof17 and AtDof1.4 were on the B3 subgroup, which was different from the previous result. There were no DhDofs that belonged to the C3 subgroup. Group E is a new branch identified for the first time. The four genes DhDof6, DhDof7, DhDof8, and DhDof9 are independent from branches A and D2. In addition, the ML phylogenetic tree of the two species was also constructed (Figure 2B). The only difference between two phylogenetic trees was that AtDof1.7 and AtDof3.1 were classified in the D2 subgroup. The properties and subcellular localization of *Dof* genes were further predicted (Table 1). Subcellular localization prediction showed that all the *DhDofs* were localized in the nucleus.

**TABLE 1:** The classification and physical properties of *Dof* genes in *D. huoshansense*

Gene ID	Gene name	Subgroup	Amino acid	Molecular weight (Da)	Theoretical pI	Instability index	Aliphatic index	Hydropathicity	Subcellular localization
*Dhu000021317*	*DhDof3*	A	271	29466.54	9.06	62.45	50.11	−0.733	nucleus
*Dhu000013776*	*DhDof11*	B1	336	35618.69	8.9	54.12	55.77	−0.489	nucleus
*Dhu000027194*	*DhDof10*	B1	336	35609.68	8.9	52.58	55.77	−0.49	nucleus
*Dhu000009040*	*DhDof14*	B2	276	29365.58	8.9	46.45	52.07	−0.5	nucleus
*Dhu000025304*	*DhDof15*	B2	276	29365.58	8.9	46.45	52.07	−0.5	nucleus
*Dhu000003320*	*DhDof4*	B3	259	27894.13	8.42	56.27	59.69	−0.42	nucleus
*Dhu000003330*	*DhDof5*	B3	259	27880.1	8.43	55.8	58.92	−0.412	nucleus
*Dhu000023804*	*DhDof17*	B3	188	20965.46	9.02	41.29	47.29	−0.494	nucleus
*Dhu000022280*	*DhDof1*	C1	275	30536.9	8.77	57.17	51.05	−0.75	nucleus
*Dhu000011377*	*DhDof22*	C2.1	219	23517.12	9.71	50	49.5	−0.7	nucleus
*Dhu000024679*	*DhDof21*	C2.1	311	34294.12	9.55	58.72	56.11	−0.803	nucleus
*Dhu000014864*	*DhDof13*	C2.2	221	25293.61	8.13	57.22	57.38	−0.685	nucleus
*Dhu000000352*	*DhDof20*	D1	423	46631.83	6.57	46.5	59.01	−0.801	nucleus
*Dhu000005270*	*DhDof16*	D1	108	11964.48	8.69	58.53	56.11	−0.58	nucleus
*Dhu000011214*	*DhDof2*	D1	553	60447.44	8.71	43.86	67.58	−0.479	nucleus
*Dhu000025282*	*DhDof12*	D1	115	12404.13	9.8	32.07	49.3	−0.477	nucleus
*Dhu000011533*	*DhDof18*	D2	245	26636.65	8.6	44.57	60.61	−0.399	nucleus
*Dhu000024068*	*DhDof19*	D2	183	19855.96	7.64	46.62	54.48	−0.59	nucleus
*Dhu000008503*	*DhDof7*	E	358	37361.93	8.59	58.77	63.63	−0.32	nucleus
*Dhu000015767*	*DhDof8*	E	203	21772.64	8.91	65.98	61.97	−0.492	nucleus
*Dhu000015769*	*DhDof6*	E	358	37355.92	8.59	60.39	62.54	−0.337	nucleus
*Dhu000023942*	*DhDof9*	E	203	21758.61	8.91	68.14	61.03	−0.503	nucleus

### Conserved domain, conserved motif and exon/intron composition of *Dof* genes

**FIGURE 3:**
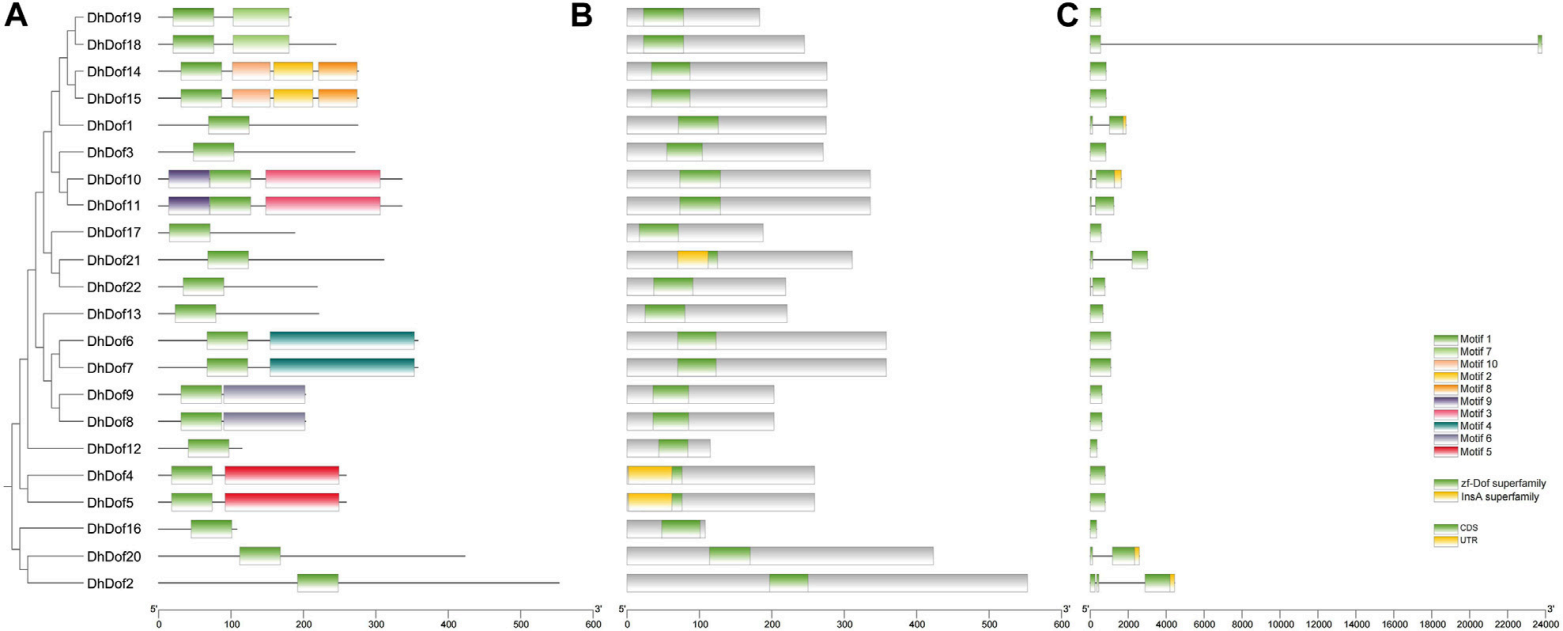
Conserved motif and exon/intron analysis of the *DhDof* gene. **(A)** Conserved motif; **(B)** conserved domain; **(C)** cds, introns and UTR.

**FIGURE 4:**
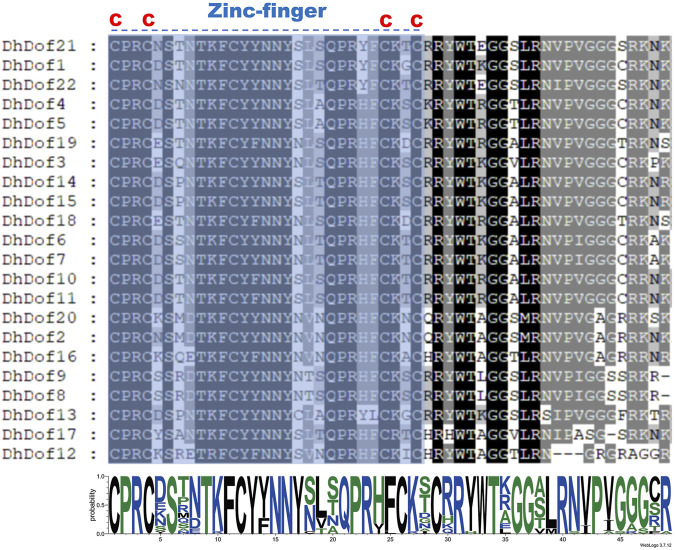
Multiple sequence alignments of the Dof protein. “C” stands for cysteine. The dashed line represents the zinc-finger motif of the Dof domain.

To clarify the gene structure and composition of different types of Dof genes, the number and composition of motifs of DhDofs were first analyzed. Motif 1 was a conserved zinc-finger motif common to all Dof genes (Figure 3A). For the same subgroup, their conserved motif composition was the same, like DhDof10 and DhDof11, which both contained motif 1, motif 3 and motif 9. DhDof14 and DhDof15 included motif 1, motif 2, motif 8 and motif 10. The conserved domain database search results showed that all DhDofs contained the zf-Dof superfamily domains, while the N-ternimus of DhDof4, DhDof5 and DhDof21 also contained an InsA superfamily domain (Figure 3B). Gene structure analysis revealed that the number and composition of introns and exons in different *DhDof* genes varied greatly. Some *DhDofs* (such as *DhDof2*, *DhDof20*, *DhDof10* and *DhDof1*) also contained 3’ UTR regions (Figure 3C). There was a long intron insertion between the two exons of DhDof18. The zinc finger structure in Dof domain is required to recognize AAAG/CTTT elements. A sequence comparison of the Dof proteins revealed that DhDofs contained a conserved CPRC(X)21CK(X)1C motif, which formed part of the Dof domain (Figure 4).

### Comparative genomic analysis of *Dof* genes

**FIGURE 5:**
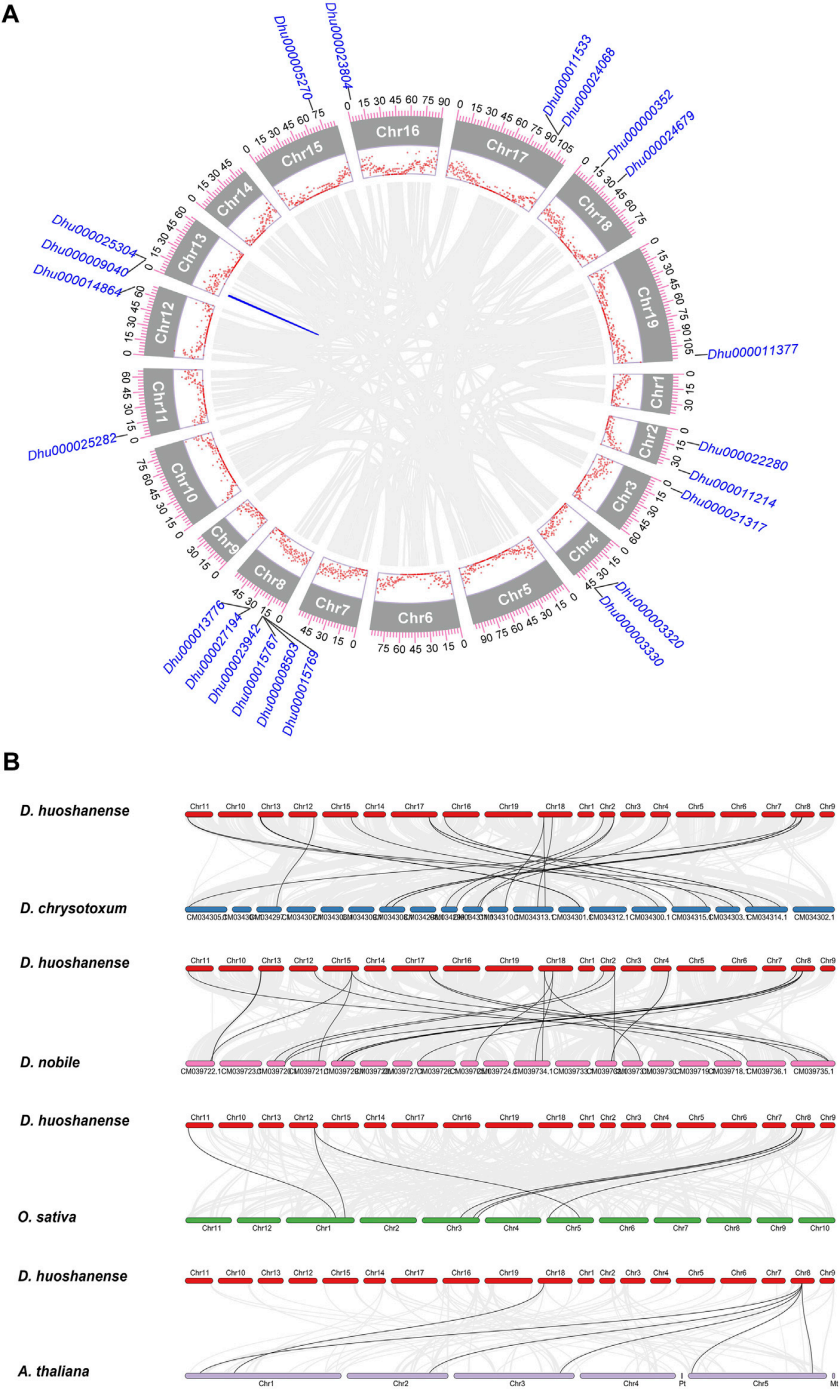
Comparative genomic analysis of *Dof* genes. **(A)** Microsynteny of *DhDof* genes; **(B)** Collinearity of *D. huoshanense* with *D. nobile*, *D. chrysotoxum*, *O. sativa* and *A. thaliana*. The ticks and scatter points are the genome scale and gene density, respectively. Gray lines represent shared gene pairs.

Comparative genomics analysis provided clues for the evolutionary relationships among species of *D. spp*. To clarify the genetic relationship of *Dof* genes between *D. huoshanense* and its closely related species, a collinearity analysis of *Dof* genes was employed. Microsynteny analysis showed that out of all the *DhDof* genes, only one pair of genes (*Dhu000025304* and *Dhu000009040*) had collinearity, and dispersed and proximal duplication contributed to the expansion of *DhDof* genes. This implied that the formation of *DhDof* homologs was not due to whole genome duplication (Figure 5A). Synteny analysis from interspecies reveals that *D. huoshanense* and *D. nobile* exhibited the highest level of collinearity, with a total of 21 gene pairs, followed by *D. huoshanense* and *D. chrysotoxum*. However, there were only 7 and 6 syntenic blocks compared with *O. sativa* and *A. thaliana*, respectively. This was in accordance with the species’ relationship (Figure 5B). Artificial selection pressure provides the driving force for genome evolution and domestication. Through calculating Ka and Ks values, the Ka/Ks ratios of all *DhDof* genes were shown less than 1, which meant that *DhDof* genes were subject to purifying selection (Supplementary Table S1).

### Expression profile and correlation analysis of *Dof* genes

**FIGURE 6:**
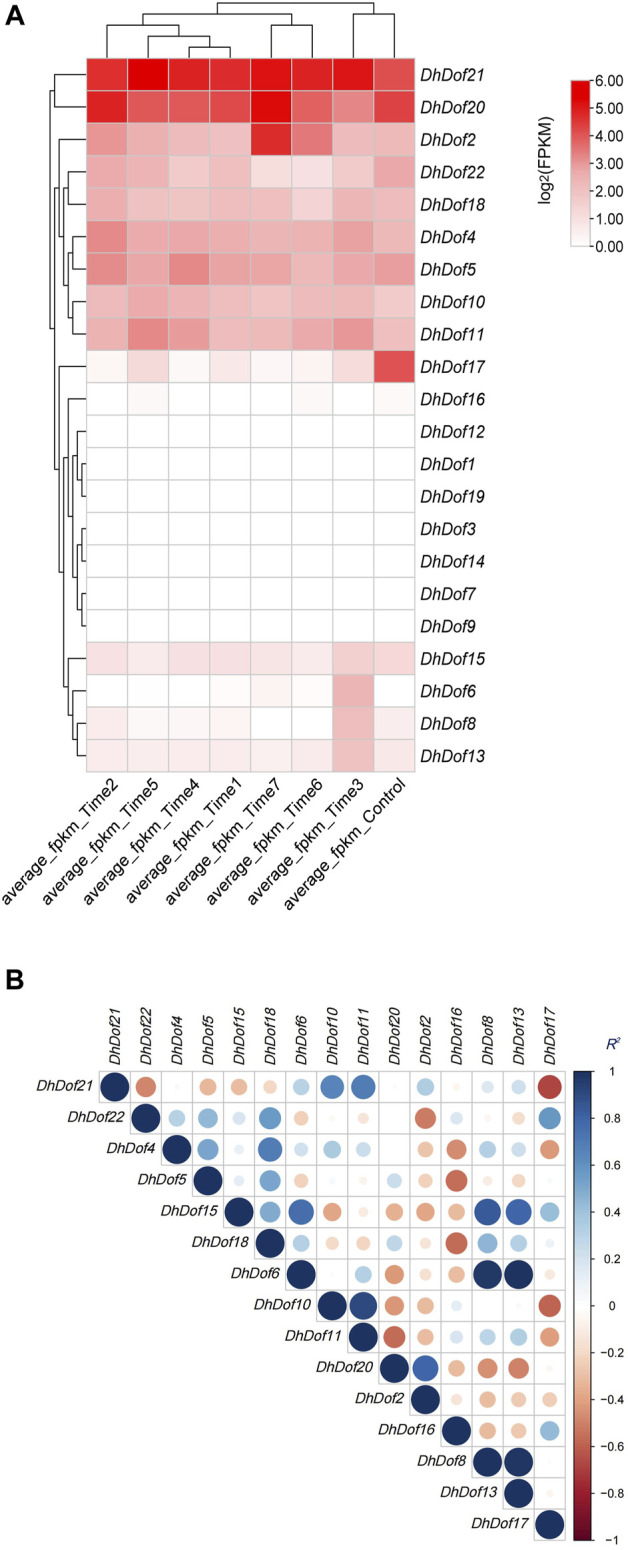
Expression profile and correlation analysis of the *DhDof* gene. **(A)** Expression profile after MeJA treatment; **(B)** correlation analysis of *DhDof* genes. Mean_Control, Mean_T1, Mean_T2, Mean_T3, Mean_T4, Mean_T5, Mean_T6, and Mean_T7 represent the groups treated with MeJA after 0, 0.25, 0.5, 1.0, 2.0, 4.0, 8.0, and 16.0 h, respectively.

Based on the transcriptome sequencing and annotation results, the expression profile of *Dofs* was examined under MeJA treatment. Almost half of the *DhDof* genes were not expressed, and only a few genes were highly expressed after treatment (Figure 6A). *DhDof20* and *DhDof21* were more sensitive to MeJA stimulation and showed high expression throughout the entire period. This suggests that they are the core JA-responsive genes and participate in the downstream regulation of JA signaling. The expression of *DhDof2* continued to increase with treatment time. The expression level reached its maximum at Time 7, showing a delayed JA-induced effect. On the contrary, the expression of *DhDof17* was the highest at the beginning and decreased significantly after MeJA treatment, which suggested that *DhDof17* was a negative effector gene of JA signaling. The expressions of *DhDof6*, *DhDof8*, and *DhDof13* were highest at Time 3 with MeJA treatment and then gradually decreased, indicating that these *DhDofs* were early-responsive genes in JA signaling. To find out which *Dof* genes are co-expressed, a correlation analysis was performed based on their expression levels. *DhDof6* showed a significantly positive correlation with *DhDof8* and *DhDof13*. *DhDof8* and *DhDof13* also showed a significantly positive correlation, followed by *DhDof15*. *DhDof6*, *DhDof8*, and *DhDof13* were co-expressed during MeJA treatment (Figure 6B). In addition, the expression of *DhDof21* and *DhDof17* showed a significant negative correlation, which was consistent with the previous result.

### *Cis*-acting element analysis of *Dof* genes

**FIGURE 7:**
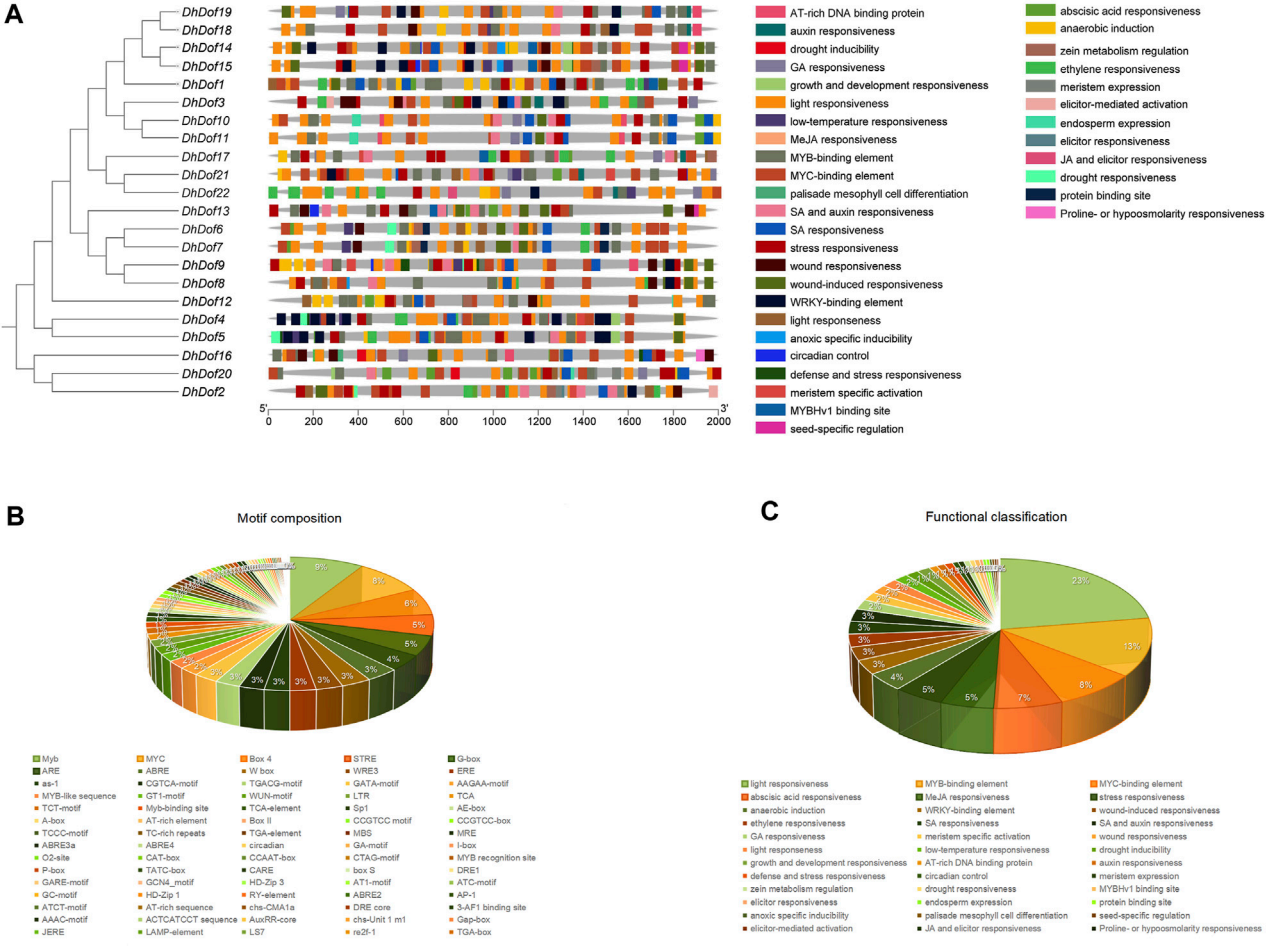
Analysis of *cis*-acting elements of the *DhDof* gene. **(A)** Distribution and composition of elements on the promoter of the *DhDof* gene; **(B)** Number and proportion of elements in the *DhDof* gene; **(C)** Functional classification and proportion of the *DhDof* gene.

To determine which kind of environmental stresses these *Dof* genes could respond to, the composition of *cis*-acting elements was analyzed. The *DhDof* gene contained numerous types of regulatory elements. *DhDofs* from the same branch or subgroup contained similar *cis*-acting elements but differed in the distribution of seldom elements. For instance, *DhDof18* contained a wound-induced element at the 5′end, but not in *DhDof19*. *DhDof19* contained a GA-responsive element at the 5′end, but not in *DhDof18*. A total of 37 functional classifications were present here, which were mainly involved in growth, hormone induction, and abiotic stress response (Figure 7A). The most distributed element was related to light responsiveness, which was present in all *DhDof* genes. Among them, *DhDof2*, *DhDof14*, and *DhDof21* were *Dof* genes that contained more functional categories. The second most common component was the MYB-binding element. *DhDof1*, *DhDof12*, and *DhDof16* contained a large number of these elements. The third common component is the MYC-binding element. A total of 76 different *cis*-acting elements were discovered, with Myb, MYC, and Box4 elements being the most prominent (Figure 7B). The regulatory elements related to plant growth and development mainly included GCN4_motif, circadian, A-box, CAT-box, CCGTCC motif, re2f-1, HD-Zip 1 and RY-element. *Cis*-acting elements involved in hormone responses were deferentially distributed in the *DhDof* genes. *DhDof2* and *DhDof17* were the key genes involved in ABA responsiveness. *DhDof21* was the key gene involved in GA responsiveness. *DhDof2* and *DhDof13* were the key genes involved in MeJA responsiveness. *DhDof10* and *DhDof11* were the key genes involved in SA responsiveness. *DhDof22* was the key gene involved in ethylene responsiveness. The *cis*-acting elements involved in abiotic stress mainly included ARE, GC-motif, TC-rich repeats, MBS, DRE1, DRE core, LTR, STRE, WUN-motif, and WRE3, which were relevant to drought, low temperature, defense stresses, anaerobic induction, mechanical injury, *etc*. (Figure 7C).

### Expression pattern analysis of *Dof* genes

**FIGURE 8:**
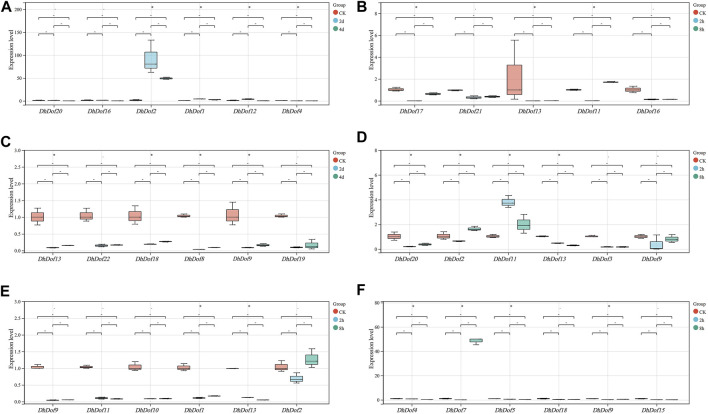
*q*RT-PCR analysis of the *DhDof* gene. **(A)** ABA treatment; **(B)** GA treatment; **(C)** IAA treatment; **(D)** MeJA treatment; **(E)** SA treatment; **(F)** low temperature treatment.

The expression pattern of some of *Dofs* was confirmed by *q*RT-PCR method. The results showed that on the second and fourth days of ABA treatment, the expression of *DhDof2* dramatically increased by approximately 92-fold and 49-fold compared with the control, respectively. *DhDof2* was the main ABA-responsive gene and could be strongly induced. The expression levels of DhDof1 and DhDof12 exhibited an initial increase followed by a later drop under ABA treatment. The expression of *DhDof4* decreased significantly after ABA treatment (Figure 8A). The expression level of *DhDof13* decreased significantly at the 2nd hour after GA treatment and remained at a low level at 8 h. The expressions of *DhDof21* and *DhDof16* were also inhibited by GA. However, the expression of *DhDof11* and *DhDof17* decreased sharply at the 2nd hour of GA treatment but basically returned to the initial level at the 8th hour (Figure 8B). IAA treatment could significantly inhibit the expression of all *DhDof* candidates, and this inhibitory effect persisted until day 4. TGA-element (AACGAC) and AuxRR-core element (GGTCCAT) in the promoter region of *DhDof* were the negative auxin-responsive elements (Figure 8C).

## Discussion

### Molecular characterization and evolution of *DhDof* genes

Dof TFs are ubiquitously found across the full spectrum of the plant kingdom, encompassing both lower plants, such as unicellular green algae, and higher plants, like angiosperms and gymnosperms (Yu et al., 2020; Song et al., 2024). *D. huoshanense* did not have as many *Dof* genes as other angiosperms, which meant it probably had not experienced large-scale genome duplication (Melgar and Zelada, 2021). *DhDofs* were unevenly distributed on 12 pseudochromosomes and not equivalent to chromosome size (Figure 1). We infer this result from the unequal gene duplications of chromosomal segments. Microsynteny analysis revealed that only one pair of *DhDof* genes had collinearity, which proved that other duplication may be relevant (Figure 5). In addition to the *Dof* gene, some other transcription factors, like bHLH and WRKY, have been shown shared syntenic blocks among *D. huoshanense*, *D. chrysotoxum*, and *D. nobile*. This provided evidence for their close genetic relationship (He et al., 2023; Pan et al., 2023). *D. huoshanense* and *D. nobile* had the most collinear gene pairs, followed by *D. huoshanense* and *D. chrysotoxum* (Figure 5). This result was consistent with the other monocots, such as *Z. mays*, *Brachypodium distachyon*, *Saccharum officinarum*, etc. (Zou and Sun, 2023).

### Phylogenetic tree, exon/intron structure and conserved motif analysis of *DhDof* genes

Phylogenetic analysis helps us understand the evolution and genetic relationship between Dof genes and Dof in other species. Based on the composition of domains and motifs, Dof genes are usually divided into four groups: A, B, C, and D. The B, C, and D groups can be further divided into several subgroups. There are also some studies that classify Dof genes into groups I-VII, which are obtained through domains and motifs. For example, 30 Dof proteins of *O. sativa* were divided into four groups and further subdivided into seven subgroups by constructing an unrooted phylogenetic tree with Arabidopsis, sorghum, and maize (Khan et al., 2021). Dof gene family was divided into six subfamilies (Group A-F) in sorghum, of which Group B contained only AtDof4.2, AtDof4.3, AtDof4.4, and AtDof4.5. 96 *Dof* genes from the wheat genome were divided into five subfamilies (Groups A-E), but only four AtDofs were categorized in Group A. Our results indicated that these four AtDofs were classified into the C3 subgroup, and no DhDof was assigned to this subgroup (Figure 2). Based on the phylogenetic tree, DhDof genes were divided into 11 subgroups, among which B3 and E subgroups are new branches identified (Figure 2). In addition to the E group, the D1 subgroup, also known as the CDF subfamily, contains the most *Dof* genes, including *DhDof2*, *DhDof20*, *DhDof12*, and *DhDof16*. AtDof1.4 was classified into Group IV or B2 subgroups in previous studies (Yanagisawa, 2002; Lijavetzky et al., 2003; Zhang et al., 2023). However, the phylogenetic trees in our study indicated that AtDof1.4 did not group with AtDof1.7, AtDof3.1, AtDof3.4, and AtDof5.8 in the D2 subgroup but as an independent subgroup. Three DhDof genes also belong to this subgroup (Figure 2). Remarkably, the E group, to which DhDof6/7/8/9 belonged, was a new branch that originated from the paralogs in D1. All the DhDof proteins possessed the highly conserved zinc-finger Dof domain (Figures 3, 4). Gene structure analysis revealed notable variations among distinct subgroups, whereas similar structures were observed in the common subgroup, as demonstrated in the motif analysis (Figure 3). Some subgroups contained their own unique motifs. Studies indicated that the introns of *IbDof* genes were relatively small, and most genes had only one intron or even no intron. Such intron-free genes may play a role in the accelerated stress response.

### Expression profiling and functional prediction of *DhDof* genes

The involvement of *Dof* genes in response to biotic stress has been well documented. However, the regulatory function of *Dof* on abiotic stress responses was only reported in a limited number of plants (Zhang et al., 2018). *RcDOFs* expressed at two distinct levels varied in response to ABA (Waschburger et al., 2022). *CmDOFs* had a role in the response to ABA and SA, which contributed to differential expression patterns. Exogenous ABA specifically dramatically increased the expression of *CmDOF12* and *CmDOF20*. *OsDOF15* mediated the growth of the main root in rice when exposed to high salinity by releasing ethylene (Qin et al., 2019). The expressions of *OsDof1* and *OsDof19* increased at low temperatures in the cold-tolerant cultivar. Overexpression of *OsDof1* resulted in a greater seed setting rate (Liu et al., 2021). An investigation and analysis of *DhDofs* were conducted in several abiotic stresses. Expression profiles showed the spatiotemporal profile of *DhDofs* after MeJA induction. *DhDof20* and *DhDof21* were identified as the main responsive genes of JA signaling, and *DhDof17* was considered to be an early-responsive gene (Figure 6). *DhDofs* showed distinct expression patterns under different abiotic stresses. Under a series of abiotic stresses, the expression patterns of the *DhDofs* were determined at different phases. *DhDof2* was strongly induced by ABA, while *DhDof7* was significantly induced by low temperatures. The expression of most *DhDof* genes showed an inhibitory state after treatments. Most genes were inhibited after IAA and SA treatment, which were probably related to the *cis*-acting elements in their promoter (Figures 7, 8). Our results revealed how *Dof* genes work and how *Dendrobium* plants interact and coordinate with the external environment.

## Conclusion

Dof TFs play multiple roles in physiological processes that involved in biotic or abiotic stresses. Twenty-two *Dof* genes were dispersed on twelve pseudochromosomes. Microsynteny analysis revealed that *DhDof* genes were not generated from large-scale gene duplication. Interspecies synteny analysis suggested that these *Dof* genes originated from a common ancestral homolog (Song et al., 2023). The syntenic blocks between *D. huoshanense*, *D. chrysotoxum*, and *D. nobile* were larger. Selection pressure analysis further revealed *DhDof* genes underwent purifying selection. Multiple sequence alignments showed that all Dof genes contain highly conserved zinc finger motif. The phylogenetic analysis divided 22 DhDof into 11 subgroups. The E group is a new branch assigned and contained four genes, including DhDof6, DhDof7, DhDof8 and DhDof9. In addition to the zf-Dof subfamily domain, a few Dof genes also contained the Ins A subfamily domain, which was thought to be related to specific regulatory functions. However, the exon/intron analysis reflected that even *Dof* genes from the same subgroup would have widely differing intron and 3′or 5′UTR. Expression profiling analysis showed that *DhDof21* and *DhDof22* were the main JA-responsive genes. The promoter of *Dof* genes has numerous hormone response-related elements, such as the CGTCA-motif, the TGACG-motif, as-1, TCA, CARE, P-box, and others. This makes them better able to sense changes in external hormones. *q*RT-PCR analysis showed that *DhDof* genes had different expression patterns in response to different abiotic stresses. As there is some variation in the number of *Dof* genes among various species, the potential functional redundancy of these genes needs to be validated by developing several knockout lines and other approaches. *Dofs* with the potential to raise crop yields can provide promising opportunities for the advancement of the food manufacturing and biofuel production.

## Data Availability

The datasets presented in this study can be found in online repositories. The names of the repository/repositories and accession number(s) can be found in the article/Supplementary Material.
